# Tuberculosis and Immune Reconstitution Inflammatory Syndrome in Patients With Inflammatory Bowel Disease and Anti-TNFα Treatment: Insights From a French Multicenter Study and Systematic Literature Review With Emphasis on Paradoxical Anti-TNFα Resumption

**DOI:** 10.1093/ofid/ofae327

**Published:** 2024-06-17

**Authors:** Ariane Amoura, Thomas Frapard, Xavier Treton, Laure Surgers, Laurent Beaugerie, Matthieu Lafaurie, Jean Marc Gornet, Raphaël Lepeule, Aurélien Amiot, Etienne Canouï, Vered Abitbol, Antoine Froissart, Mathias Vidon, Yann Nguyen, Agnès Lefort, Virginie Zarrouk

**Affiliations:** Service de Médecine Interne, Hôpital Beaujon, Assistance Publique des Hôpitaux de Paris, Clichy, France; Sorbonne Université, Paris, France; Service de Médecine Intensive et Réanimation, Hôpital Henri Mondor, DHU ATVB, Assistance Publique des Hôpitaux de Paris, Créteil, France; Faculté de Médecine de Créteil, Université Paris Est Créteil, Institut Mondor de Recherche Biomédicale-Groupe de Recherche Clinique CARMAS, Créteil, France; Institut des MICI, Groupe hospitalier privé Ambroise-Paré-Hartmann, Neuilly, France; Service des Maladies Infectieuses et Tropicales, Hôpital Saint-Antoine, Assistance Publique des Hôpitaux de Paris, Sorbonne Université, Paris, France; Sorbonne Université, INSERM, Institut Pierre Louis d’Épidémiologie et de Santé Publique, Paris, France; Service de Gastroentérologie, Hôpital Saint Antoine, Assistance Publique des Hôpitaux de Paris, Paris, France; Service de Maladies infectieuses et Tropicales, Hôpital Saint-Louis-Hôpital Lariboisière, Assistance Publique des Hôpitaux de Paris, Paris, France; Service de Gastroentérologie, Hôpital Saint-Louis-Hôpital, Assistance Publique des Hôpitaux de Paris, Paris, France; Unité Transversale de Traitement des Infections, Assistance Publique des Hôpitaux de Paris, Hôpitaux Universitaires Henri Mondor, Créteil, France; Service de Gastroentérologie, Hôpitaux Universitaires Henri Mondor, Assistance Publique des Hôpitaux de Paris, Créteil, France; Équipe Mobile d’Infectiologie, Assistance Publique des Hôpitaux de Paris, APHP-CUP, Hôpital Cochin, Paris, France; Service de gastroentérologie, Hôpital Cochin, Assistance Publique des Hôpitaux de Paris, Université Paris Cité, Paris, France; Service de Médecine interne, Centre Hospitalier Intercommunal de Créteil, Créteil, France; Service de Gastroentérologie, Centre Hospitalier Intercommunal de Créteil, Créteil, France; Service de Médecine Interne, Hôpital Beaujon, Assistance Publique des Hôpitaux de Paris, Clichy, France; Centre de recherche en immunologie des maladies, INSERM U1184, Université Paris Saclay, Le Kremlin-Bicêtre, France; Service de Médecine Interne, Hôpital Beaujon, Assistance Publique des Hôpitaux de Paris, Clichy, France; Groupe de recherche Infection Antimicrobials Modelling Evolution, Inserm U1137, Université Paris Cité, Paris, France; Service de Médecine Interne, Hôpital Beaujon, Assistance Publique des Hôpitaux de Paris, Clichy, France

**Keywords:** immune reconstitution inflammatory syndrome, infliximab, paradoxical reaction, TNF-α antagonists, tuberculosis

## Abstract

**Background:**

The advent of anti–tumor necrosis factor α (anti-TNFα) has revolutionized the treatment of inflammatory bowel disease (IBD). However, susceptibility to active tuberculosis (TB) is associated with this therapy and requires its discontinuation. The risk of immune reconstitution inflammatory syndrome (IRIS) in this population is poorly understood, as is the safety of resuming anti-TNFα.

**Methods:**

This French retrospective study (2010–2022) included all TB cases in patients with IBD who were treated with anti-TNFα in 6 participating centers. A systematic literature review was performed on TB-IRIS and anti-TNFα exposure.

**Results:**

Thirty-six patients were included (median age, 35 years; IQR, 27–48). TB was disseminated in 86% and miliary in 53%. IRIS occurred in 47% after a median 45 days (IQR, 18–80). Most patients with TB-IRIS (93%) had disseminated TB. Miliary TB was associated with IRIS risk in univariate analysis (odds ratio, 7.33; 95% CI, 1.60–42.82; *P* = .015). Anti-TB treatment was longer in this population (median [IQR], 9 [9–12] vs 6 [6–9] months; *P* = .049). Anti-TNFα was resumed in 66% after a median 4 months (IQR, 3–10) for IBD activity (76%) or IRIS treatment (24%), with only 1 case of TB relapse. Fifty-two cases of TB-IRIS in patients treated with anti-TNFα were reported in the literature, complicating disseminating TB (85%) after a median 42 days (IQR, 21–90), with 70% requiring anti-inflammatory treatment. Forty cases of TB-IRIS or paradoxical reaction treated with anti-TNFα were also reported. IRIS was neurologic in 64%. Outcome was mostly favorable (93% recovery).

**Conclusions:**

TB with anti-TNFα treatment is often complicated by IRIS of varying severity. Restarting anti-TNFα is a safe and effective strategy.

The advent of anti–tumor necrosis factor α (anti-TNFα) therapies has revolutionized the management of chronic inflammatory bowel disease (IBD) [1]. However, since the early 21st century, evidence has emerged of an inherent susceptibility to active tuberculosis (TB) associated with their administration [2]. This susceptibility is primarily due to reactivation of latent TB [3], with relative risk ranging from 2 to 16 in the literature [4–6].

This observation has led to the recommendation of screening and treating latent TB infection (LTBI) before initiating anti–tumor necrosis factor (anti-TNF) therapy [7]. In particular, TB under anti-TNF therapy manifests as aggressive forms, often extrapulmonary and disseminated [8, 9]. Unfortunately, TB under anti-TNF treatment mandates discontinuation of this therapeutic [10], even in patients with severe gastrointestinal pathology. The optimal time frame for resuming such treatment remains unclear, although several guidelines suggest a provisional period of 2 months, without clear justification [10].

The ensuing immune reconstitution associated with the cessation of anti-TNFα, coupled with the propensity for disseminated TB manifestations, creates increased susceptibility in patients to the potential onset of immune reconstitution inflammatory syndrome (IRIS), a phenomenon that can exhibit varying degrees of clinical severity [11, 12].

Management of IRIS lacks standardized protocols and is primarily dictated by the severity of its manifestations. It ranges from symptomatic treatment to predominantly anti-inflammatory approaches, often including corticosteroid therapy [13]. Paradoxically, a handful of studies report the use of anti-TNFα agents in this context [14, 15].

The aim of our study was to delineate the clinical features of TB in patients undergoing anti-TNFα therapy for IBD and to assess IRIS within this demographic, as well as its severity and subsequent management.

To achieve this objective, we conducted a retrospective multicenter study in the Ile-de-France region, France, involving patients with IBD undergoing anti-TNF therapy who experienced active TB. Additionally, we performed a systematic literature review on TB-IRIS in this population receiving anti-TNFα therapy, as well as cases of TB-IRIS treated with anti-TNFα agents.

## METHODS

### Cohort

We performed a 12-year multicenter retrospective study in 6 French teaching hospitals in Ile-de-France. All consecutive patients were included if hospitalized in the participating centers between 2010 and 2022 with proven TB disease during treatment with anti-TNFα for IBD.

Patients were identified at each center through a request for the following *ICD-10* codes: IBD (K50, K51, K52) and TB (A15–A19). Medical records were reviewed to verify the inclusion criteria: age >18 years, diagnosis of IBD, treatment with anti-TNFα at the time of TB diagnosis, and proven TB (positive culture for *Mycobacterium tuberculosis* complex or granulomatous lesion with caseous necrosis).

#### Collection of Data

Data were collected on demographics, comorbidities, clinical examination, laboratory findings, imaging findings, microbiologic testing, and therapeutic management during hospitalization and follow-up. In this research, IRIS was defined in operational terms as the appearance of new lesions or the deterioration of preexisting ones after a period of improvement, with no proof of TB recurrence or noncompliance with medication or adverse drug reactions.

#### Statistical Analysis

Quantitative data were expressed as median (IQR) according to their nonnormal distribution. Quantitative data were compared by a nonparametric Mann-Whitney test according to their distribution. Categorical data were compared by a Fisher exact test. Factors associated with IRIS were analyzed by univariate logistic regression. The tests were 2-tailed, and *P* < .05 was considered statistically significant. Statistical analysis was performed with R version 4.2.2 (R Foundation for Statistical Computing).

#### Ethical Considerations

The database is registered at the Commission Nationale de l’Informatique et des Libertés (2225933) and was approved by the Ethics Committee of the French Infectiology Society (CER-MIT 2023-0904).

The investigation conforms with the principles outlined in the Declaration of Helsinki. In accordance with the ethical standards of our hospital's institutional review board, the Committee for the Protection of Human Subjects, and French law, written informed consent was not needed for demographic, physiologic, and hospital outcome data analyses because this observational study did not modify existing diagnostic or therapeutic strategies; however, patients were informed of their inclusion in the study.

### Systematic Review

#### Search Strategy and Selection Criteria

This study was performed in accordance with the PRISMA statement (Preferred Reporting Items for Systematic Reviews and Meta-analyses) [16]. Electronic databases were searched August 2023, including MEDLINE via the OVID platform (1946–present), Embase (1980–present), Cochrane, and PubMed. The search strategies are detailed in Supplementary Table 1. Studies were screened by 2 independent reviewers in separate databases. We included all studies about TB-IRIS and anti-TNFα treatment. We focused on studies written in English or French. After the removal of the duplicates, 2 reviewers independently screened titles and abstracts to obtain relevant articles for full-text analysis. Eligible articles were then selected for the review. Any disagreement was resolved by discussion with a third reviewer. Ethical approval was not required.

## RESULTS

### Cohort

Thirty-six patients were included in this study (male, 64%; median age, 35 years [IQR, 27–48]; Table 1). Thirty-one (86%) had Crohn disease and 5 (14%) had ulcerative colitis. The median duration of anti-TNFα therapy was 8 months (IQR, 2–24). Sixteen patients had early TB (44%; within 6 months after anti-TNFα was started) and 10 had late TB (28%; >18 months). Twenty patients (57%) received combination therapy with anti-TNFα and azathioprine.

**Table 1.** Demographic and Clinical Characteristics of 36 Patients Treated With Anti-TNFα Antibodies at the Time of TB Diagnosis

	Median (IQR) or No. (%)
Demographics	
Age, y	35 (27–48)
Male	23 (63.9)
Smoking	15 (45.5)
IBD	
Crohn disease	31 (86.1)
Ulcerative colitis	5 (13.9)
Time since IBD diagnosis, mo	84 (48–132)
Ongoing treatment	
Anti-TNFα antibody	36 (100)
Infliximab	23 (63.9)
Adalimumab	13 (36.1)
Anti-TNFα duration, mo	8 (2–24)
Azathioprine	20 (57.1)
Corticosteroids	4 (11.4)
LTBI screening and treatment	
Missing data	6 (16.7)
Tuberculosis skin tests	9 (32)
Interferon γ release assays	24 (85.7)
Chest radiograph	27 (96.4)
Thoracic tomodensitometry	2 (7.1)
No screening	3 (8.3)
LTBI diagnosis	6 (21.4)
LTBI treatment	6 (21.4)
Pulmonary TB diagnosis	1 (3.6)
Anti-TB treatment	1 (3.6)
Risk factors for TB	
HIV infection	0 (0)
Previous TB infection	1 (2.8)
Contact with patient with TB	3 (8.3)
Coming from endemic country for TB	19 (52.8)
TB features	
Hospitalization	36 (100)
Hospitalization in intensive care unit	6 (16.7)
Disseminated TB	31 (86.1)
Pulmonary	35 (97.2)
Miliary	19 (52.8)
Pulmonary condensation	18 (50)
Cavities	5 (13.9)
Tree-in-bud pattern	16 (44.4)
Intrathoracic nodes	34 (94.4)
Pleural effusion	7 (19.4)
Neuromeningeal	3 (8.3)
Pericarditis	5 (13.9)
Ascites	3 (8.3)
Hemophagocytosis	4 (11.1)
Extrathoracic lymph nodes	20 (55.6)
Hepatic or splenic	13 (36.1)
TB treatment	
Anti-TNFα withdrawal	36 (100)
Anti-TB treatment	36 (100)
Corticosteroids for CNS TB	3 (8.3)
Antibiotic duration, mo	6 (6–9)
TB-IRIS	15 (46.9)
Missing data	4
Time between TB diagnosis and IRIS	45 (18–80)
Fever	9 (60)
Aggravation of pulmonary lesions	7 (46.7)
Aggravation or onset of lymphadenopathy	9 (60)
Pancytopenia	1 (6.7)
Exacerbation of digestive inflammation	3 (20)
Exacerbation of hepatic/splenic lesions or cytolysis	3 (20)
AKI with hypercalcemia	1 (6.7)
Neurologic IRIS	1 (6.7)
C-reactive protein, mg/L	77.50 (51.5–91.2)
No specific treatment	5 (33.3)
Corticosteroids	8 (53.3)
Anti-TNFα resumption for IRIS	5 (33.3)
Cure without sequelae	15 (100)
Outcome	
Missing data	4
Follow-up, mo	31 (18–88)
TB relapse	2 (6.3)
Anti-TNFα resumption	21 (65.6)
Time before resumption, mo	4 (3–10)
Systematic	2 (9.5)
For IBD activity	16 (76.2)
For IRIS	5 (23.8)
Follow-up after resumption, mo	27 (17–82)

Abbreviations: AKI, acute kidney injury; anti-TNFα, anti–tumor necrosis factor α; CNS, central nervous system; IBD, inflammatory bowel disease; IRIS, immune reconstitution inflammatory syndrome; LTBI, latent tuberculosis infection; TB, tuberculosis.

Screening for LTBI prior to anti-TNFα initiation was negative in 20 (67%) of 30 patients; among the other 10 patients, 6 (21%) were treated for LTBI, 1 (3%) for pulmonary TB, and 3 (8%) had no screening. Among the patients with negative LTBI screening, 14 (70%) were treated with azathioprine prior to the screening. Out of 36 patients, 19 (53%) came from a TB-endemic country; 3 (8%) had been exposed to a patient with TB; and 1 (3%) had a personal history of TB. The 6 patients treated for LTBI reported TB after 20 months (IQR, 11.75-56.25)—1 of them only 2 months after treatment of latent TB, raising the suspicion of poor treatment compliance. Among the 5 others, 4 (80%) had been reexposed to the disease by contact or travel to endemic countries.

TB was disseminated in most cases (31/36, 86%) and severe in 6 (17%), requiring hospitalization in an intensive care unit. Miliary TB occurred in 19 (53%) cases, pericarditis in 5 (14%), hemophagocytosis in 4 (11%), and neuromeningeal TB in 3 (8%).

In all patients, anti-TNFα therapy was stopped at the time of TB diagnosis, and anti-TB treatment (ATT) was prescribed for a median 6 months (IQR, 6–9). IRIS occurred in 15 (47%) of 32 patients, with a median time of 45 days (IQR, 18–80) between TB diagnosis and IRIS onset. Patients had worsening of pulmonary lesions (n = 7, 47%) and/or worsening or new onset of lymphadenopathy (n = 9, 60%). Most of them also had fever (n = 9, 60%). Of 15 patients, 5 (33%) did not receive specific treatment, 8 (53%) were treated with corticosteroids, and anti-TNFα was represcribed for IRIS treatment in 5 (33%). Every patient was cured without sequelae from IRIS.

Comparison of IRIS and non-IRIS cases and univariate analysis for IRIS risk factors are shown in Table 2. Notably, the median (IQR) time since IBD diagnosis was longer in non-IRIS cases (108 [78–162] vs 48 [25–81] months, *P* = .013), but the duration of anti-TNFα treatment was comparable (12 [3–30] vs 11.50 [3–46] months). Disseminated TB was predominant in both groups (82% in non-IRIS, 93% in IRIS). Miliary TB was a risk factor for IRIS in univariate analysis with an odds ratio of 7.33 (95% CI, 1.60–42.82; *P* = .015). ATT was longer in the IRIS group (9 [9–12] vs 6 [6–9] months).

**Table 2.** Demographic and Clinical Characteristics of 17 Non-IRIS and 15 IRIS-TB Cases From the Current Cohort and Univariate Analysis for the Risk of IRIS

	Median (IQR) and No. (%)			Univariate Analysis
	Non-IRIS (n = 17)	IRIS (n = 15)	*P* Value	Odds Ratio (95% CI; *P* Value)
Demographics				
Age, y	38 (28–52)	34 (26–40)	.168	0.95 (.88–1.01; .108)
Male	9 (52.9)	13 (86.7)	.060	5.78 (1.13–44.69; .052)
Smoking	8 (53.3)	5 (35.7)	.462	0.49 (.10–2.12; .343)
IBD				
Crohn disease	14 (82.3)	13 (86.7)		…
Ulcerative colitis	3 (17.6)	2 (13.3)		…
Time since diagnosis, mo	108 (78–162)	48 (25–81)	.**013**	**0.98 (.96–1.00; .033)**
Ongoing treatment				
Anti-TNFα	17 (100)	15 (100)		…
Infliximab	11 (64.7)	10 (66.7)	>.99	…
Adalimumab	6 (35.3)	5 (33.3)	>.99	…
Anti-TNFα duration, mo	12 (3–30)	11.50 (3–46)	.913	1 (.97–1.03; .929)
Azathioprine	9 (52.9)	11 (73.3)	.291	0.33 (.02–2.97; .366)
Corticosteroids	3 (17.6)	1 (6.7)	.603	2.44 (.57–11.78; .239)
TB features				
Disseminated TB	14 (82.4)	14 (93.3)	.603	3.00 (.34–64.84; .366)
Miliary	6 (35.3)	12 (80.0)	.**016**	**7.33 (1.60–42.82; .015)**
Neuromeningeal	1 (5.9)	2 (13.3)	.589	2.46 (.21–56.53; .482)
Lymphocyte count/mm^3^	1220 (1015–1620)	820 (700–1650)	.411	1.00 (1.00–1.00; .504)
Hemoglobin, g/dL	12.30 (10.5–13.40)	11.40 (10.85–12.95)	.628	0.87 (.57–1.28; .497)
TB treatment				
Anti-TNFα withdrawal	17 (100)	15 (100)		…
Anti-TB treatment	17 (100)	15 (100)		…
Corticosteroids	1 (5.9)	2 (13.3)	.589	2.91 (.25–67.27; .406)
Antibiotic duration, mo	6.00 (6.00–9.00)	9.00 (9.00–12.00)	.**049**	1.34 (1.01–1.90; .068)

Bold indicates *P* < .05.

Abbreviations: anti-TNFα, anti–tumor necrosis factor α; IBD, inflammatory bowel disease; IRIS, immune reconstitution inflammatory syndrome; TB, tuberculosis.

Anti-TNFα was represcribed after a median 4 months (IQR, 3–10) in 21 (66%) of 32 cases, mostly for IBD activity (16/21, 76%) after 4 months (IQR, 4–12) or to treat IRIS (5/21, 24%) with dramatic efficacity. Median follow-up after anti-TNFα resumption was 27 months (IQR, 17–82), and TB relapse occurred in only 1 patient. This patient had disseminated TB and was treated with ATT for 10 months. Anti-TNF were resumed 8 months after the end of ATT for Crohn disease activity. Three months after restarting anti-TNF, he presented with disseminated pansensitive *M tuberculosis* TB (Table 1).

### Systematic Literature Review

#### Studies Characteristics

**Figure 1.**
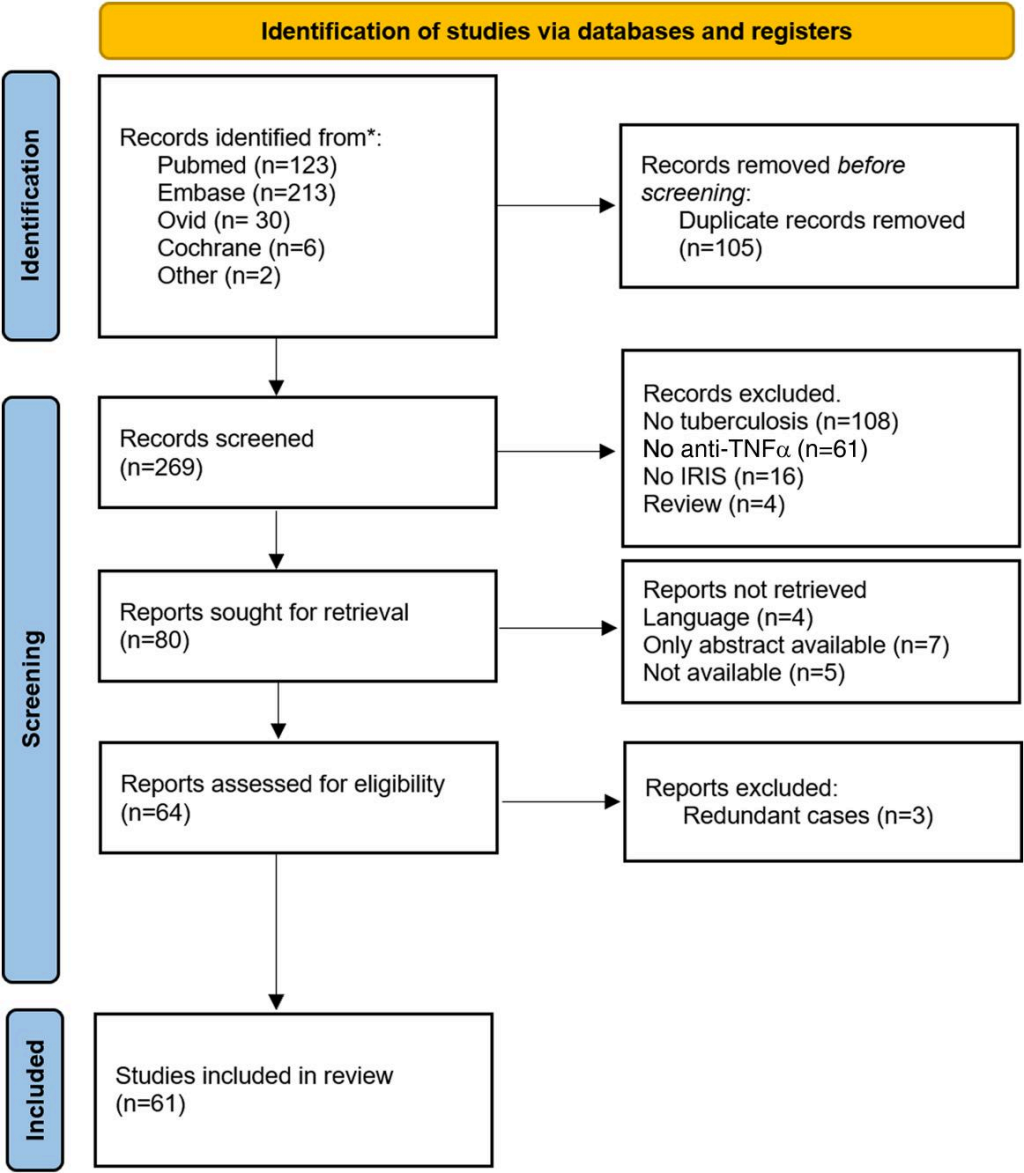
Flow diagram for the systematic literature review. From the PRISMA 2020 statement. anti-TNFα, anti–tumor necrosis factor α; IRIS, immune reconstitution inflammatory syndrome.

After removal of duplicates, 269 records were screened, and 64 were assessed for eligibility. After verification of inclusion criteria, 61 studies were selected for the systematic review (see flow diagram, Figure 1). Studies are listed in Supplementary Table 2. Most studies were case reports (n = 44) or case series (n = 11) reporting either cases of IRIS in patients treated for TB who were taking anti-TNFα agents for inflammatory/autoimmune disease or TB-IRIS cases treated with anti-TNFα. Others were articles mentioning the risk of IRIS in patients being treated with anti-TNFα.

#### IRIS in Anti-TNFα TB Cases

Sixty-seven patients were studied (65% male; median age, 41 years [IQR, 28–56]): 52 from the literature and 15 from the current study. The main characteristics are listed in Table 3. All patients were treated with anti-TNFα at the time of TB diagnosis: 42% for IBD, 20% for rheumatoid arthritis, 17% for spondyloarthropathy, 12% for psoriasis.

**Table 3.** Demographic and Clinical Characteristics of 67 Patients Who Developed TB While Undergoing Anti-TNFα Treatment and Whose TB Was Complicated by IRIS

	Median (IQR) or No. (%)
Demographics	
Age, y	41 (28–56)
Male	35 (64.8)
Underlying disease	
Crohn disease	23 (34.3)
Ulcerative colitis	5 (7.5)
Rheumatoid arthritis	12 (17.9)
Spondyloarthropathy	11 (16.4)
Psoriasis	8 (11.9)
SAPHO	3 (4.5)
Behcet disease	2 (3)
Chronic juvenile arthritis	2 (3)
Giant cell arteritis	1 (1.5)
Ongoing treatment	
Anti-TNFα	67 (100)
Infliximab	36 (53.7)
Adalimumab	28 (41.8)
Etanercept	1 (1.5)
Golimumab	1 (1.5)
Certolizumab	1 (1.5)
Anti-TNFα duration, mo	9 (2–36)
Azathioprine	12 (17.9)
Corticosteroids	9 (13.4)
Methotrexate	6 (9)
TB features	
Pulmonary tuberculosis only	4 (6)
Disseminated TB	57 (85)
Miliary	36 (53.7)
Neuromeningeal	8 (11.9)
Hemophagocytic lymphohistiocytosis	5 (7.5)
TB treatment	
Anti-TNFα withdrawal	67 (100)
Anti-TB treatment	67 (100)
Corticosteroids	13 (19.4)
TB-IRIS	
Time between TB diagnosis and IRIS, d	42 (21–90)
Intensive care unit hospitalization	2 (3)
Fever	30 (44.8)
Aggravation of pulmonary lesions	23 (34.3)
Aggravation or onset of lymphadenopathy	29 (43.3)
Aggravation of pleural or pericardial effusion	15 (22.4)
Pancytopenia	2 (3)
Exacerbation of digestive inflammation	4 (6)
Exacerbation of hepatic or splenic lesions	4 (6)
Acute kidney injury	2 (3)
Neurologic IRIS	14 (20.9)
Osteitis	1 (1.5)
Soft tissue abscess	2 (3)
TB-IRIS treatment	
Symptomatic treatment only	20 (29.9)
Corticosteroids	39 (58.2)
Anti-TNFα resumption	11 (16.4)
Thalidomide	1 (1.5)
Cyclosporine	1 (1.5)
Nonsteroidal anti-inflammatory	1 (1.5)
Methotrexate	1 (1.5)
Surgery	7 (10.4)
Outcome	
Missing data	14 (20.9)
Recovery without sequelae	46 (86.8)
Recovery with sequelae	5 (9.4)
Death from TB-IRIS	1 (1.9)
TB relapse	3 (5.7)

Patients: 52 from the systematic literature review and 15 additional cases from the current study.

Abbreviations: anti-TNFα, anti–tumor necrosis factor α; IRIS, immune restitution inflammatory syndrome; SAPHO, synovitis, acne, pustulosis, hyperostosis, and osteitis; TB, tuberculosis.

TB occurred after a median 9 months (IQR, 2–36) of anti-TNFα treatment and was disseminated in most cases (85%). Thirty-six (54%) patients had miliary TB, and 8 (12%) had central nervous system TB. Anti-TNFα was discontinued in all patients at TB diagnosis, and all patients received ATT.

The median time between TB diagnosis and IRIS was 42 days (IQR, 21–90). Two patients required intensive care unit admission and orotracheal intubation for TB-IRIS with acute respiratory distress. Patients presented with fever (45%), worsening or new onset of lymphadenopathy (43%), and worsening of pulmonary lesions (34%). Fourteen patients (21%) had neurologic IRIS. Of note, 2 had acute kidney injury attributed to IRIS.

Twenty patients (30%) received symptomatic treatment only (paracetamol, nonsteroidal anti-inflammatory) or no treatment, with a favorable outcome. Thirty-nine (58%) patients were treated with corticosteroids and 16% with anti-TNFα.

Most patients (87%) recovered without sequelae. A 75-year-old female patient died. She had disseminated TB with miliary involvement. Three weeks after starting ATT, she presented with respiratory distress due to worsening of pulmonary lesions and was intubated. Although a paradoxical reaction was diagnosed (with very high serum TNF levels), she did not receive specific treatment, and her pulmonary lesions progressed to fibrosis and death [17]. TB relapse occurred in 2 cases, 1 of which was previously described in our cohort. The second had enlargement of cervical adenopathy attributed to IRIS and was treated surgically. He received ATT for 9 months, and despite a complete recovery and no resumption of anti-TNF, he relapsed 10 months later with abdominal TB [12].

#### IRIS Treated With Anti-TNFα

Forty-five patients were studied (49% male; median age, 34.5 years [IQR, 27–44]): 40 from the literature and 5 from the current study. Main characteristics are detailed in Table 4.

**Table 4.** Demographic and Clinical Characteristics of 45 Patients Treated With Anti-TNFα for TB-IRIS

	Median (IQR) or No. (%)
**Demographics**	
Age, y	34.5 (27–43.5)
Male	22 (48.9)
Coming from endemic country for TB	21 (46.7)
Immunodepression factor	
None	24 (53.3)
HIV	9 (20)
CD4 level at TB diagnosis	124 (106–147)
Anti-TNFα treatment	11 (24.4)
Inflammatory bowel disease	7 (15.6)
Rheumatoid arthritis	1 (2.2)
Spondyloarthropathy	1 (2.2)
Psoriasis	1 (2.2)
Chronic juvenile arthritis	1 (2.2)
Treatment with mycophenolate mofetil	1 (2.2)
TB features	
Pulmonary tuberculosis only	3 (6.7)
Disseminated	29 (64.4)
Miliary	16 (35.6)
Neuromeningeal	25 (55.6)
TB treatment	
Anti-TB treatment	45 (100)
Corticosteroids	26 (57.8)
TB-IRIS or paradoxical reaction features	
Time between TB diagnosis and IRIS, d	36 (21–63)
Neuromeningeal IRIS	29 (64.4)
Aggravation of pulmonary lesions	6 (13.3)
Aggravation or onset of lymphadenopathy	10 (22.2)
Spondylodiscitis	3 (6.7)
Psoas abscess	2 (4.4)
Chylothorax	1 (2.2)
Intra-abdominal collection	1 (2.2)
Pancytopenia	1 (2.2)
Uveitis	1 (2.2)
IRIS treatment	
Intensive care unit hospitalization	5 (11.1)
Corticosteroids	42 (93.3)
Anti-TNFα	45 (100)
Infliximab	37 (82.2)
Adalimumab	9 (20)
Outcome	
Recovery without sequelae	28 (62)
Recovery with sequelae	15 (33.3)
Treatment failure	1 (2.2)
Death from IRIS	1 (2.2)

Patients: 40 from systematic literature review and 5 additional cases from the current study.

Abbreviations: anti-TNFα, anti–tumor necrosis factor α; IRIS, immune reconstitution inflammatory syndrome; TB, tuberculosis.

Twenty-one patients were immunocompromised at the time of TB diagnosis (9 had HIV, 11 were treated with anti-TNFα, and 1 with mycophenolate mofetil), whereas 24 (53%) were immunocompetent. Most patients had disseminated (64%), miliary (36%), or central nervous system (56%) TB. With ATT treatment, 58% of patients received corticosteroids.

IRIS occurred at a median 36 days (IQR, 21–63) after TB diagnosis. Most patients with IRIS (64%) had neurologic tropism. Six patients had worsening of pulmonary lesions. Three patients (7%) had worsening or new-onset spondylodiscitis, and 2 (4%) had psoas abscess. Most patients (93.3%) were treated with corticosteroids in the first instance, and all received anti-TNFα treatment (87% infliximab and 20% adalimumab) for IRIS for corticosteroid failure, due to relapse during corticosteroid tapering, or as corticosteroid-sparing treatment.

Twenty-eight patients (62%) recovered without sequelae and 33% with sequelae. One patient with central nervous system TB and IRIS did not improve despite corticosteroids and anti-TNF and remained in a deep coma; after 4 months of treatment, palliative care was decided upon and she died few days after [18]. Psoas abscess persisted despite anti-TNFα treatment in 1 patient.

Manesh et al recently performed a matched retrospective cohort study to evaluate neurologic improvement in patients with central nervous system TB receiving corticosteroids alone or anti-TNF. Forty of 90 patients had a paradoxical reaction, 19 of whom were treated with infliximab. Overall, infliximab treatment was the only parameter associated with disability-free survival at 6 months [15].

## DISCUSSION

In this study, we describe 36 cases of TB in patients receiving anti-TNFα to treat IBD in Ile-de-France, the region with the highest prevalence of TB in metropolitan France [19]. We confirmed that in patients treated with anti-TNFα, TB cases are characterized by few isolated pulmonary forms but rather disseminated forms, as noted in several previous studies [2, 4]. A notable result is the occurrence of IRIS in our patients, with 15 of 32 experiencing it. This number is higher than the 7% reported in the RATIO cohort [11], in a different study population with more rheumatologic diseases, raising the question of a potentially higher proportion of IRIS in IBD or granulomatous diseases (eg, Crohn disease). Only 1 patient had sarcoidosis in our literature review, but anti-TNFα prescription is less common in this indication. However, in the Abitbol et al study on TB exclusively in patients with IBD, only 1 case of IRIS was reported out of 44 patients [20]. The only clearly identified risk factor in univariate analysis for IRIS was the presence of miliary TB, with an odds ratio of 7.33. Rivoisy et al also noted the prescription of corticosteroids for TB and disseminated forms [11]. We did not find these results. Yet, the majority of patients in our cohort had a disseminated form of TB at diagnosis, and very few received corticosteroids.

We aimed to study IRIS under anti-TNFα more precisely and conducted a systematic literature review, which included 52 cases in addition to the 15 cases in our cohort. This review indicates that IRIS appears to be prevalent in this population, with presentations of varying severity, ranging from increased lymphadenopathy to marked general symptoms with exacerbation of pulmonary lesions potentially leading to respiratory failure. In cases with mild symptoms, given the spontaneously favorable course, symptomatic treatment only seems justified [21]. Corticosteroids appear effective in cases that require treatment in a majority of situations, although there is a risk of relapse upon tapering or corticosteroid dependency. In cases of corticosteroid therapy failure or the most severe cases, resuming anti-TNFα treatment could be an interesting option. In the literature, 40 TB-IRIS cases (plus 5 in our cohort) were treated with anti-TNFα, with favorable outcomes in most cases. Interestingly, the majority of patients had severe neurologic forms of IRIS. A recent Indian cohort study confirmed the benefits of anti-TNFα in neurologic IRIS [15]; in fact, the prescription of infliximab was the only protective factor in the multivariate analysis for neurologic prognosis at 6 months. No adverse effects related to anti-TNFα prescribing have been reported in our patients or in the literature.

**Figure 2.**
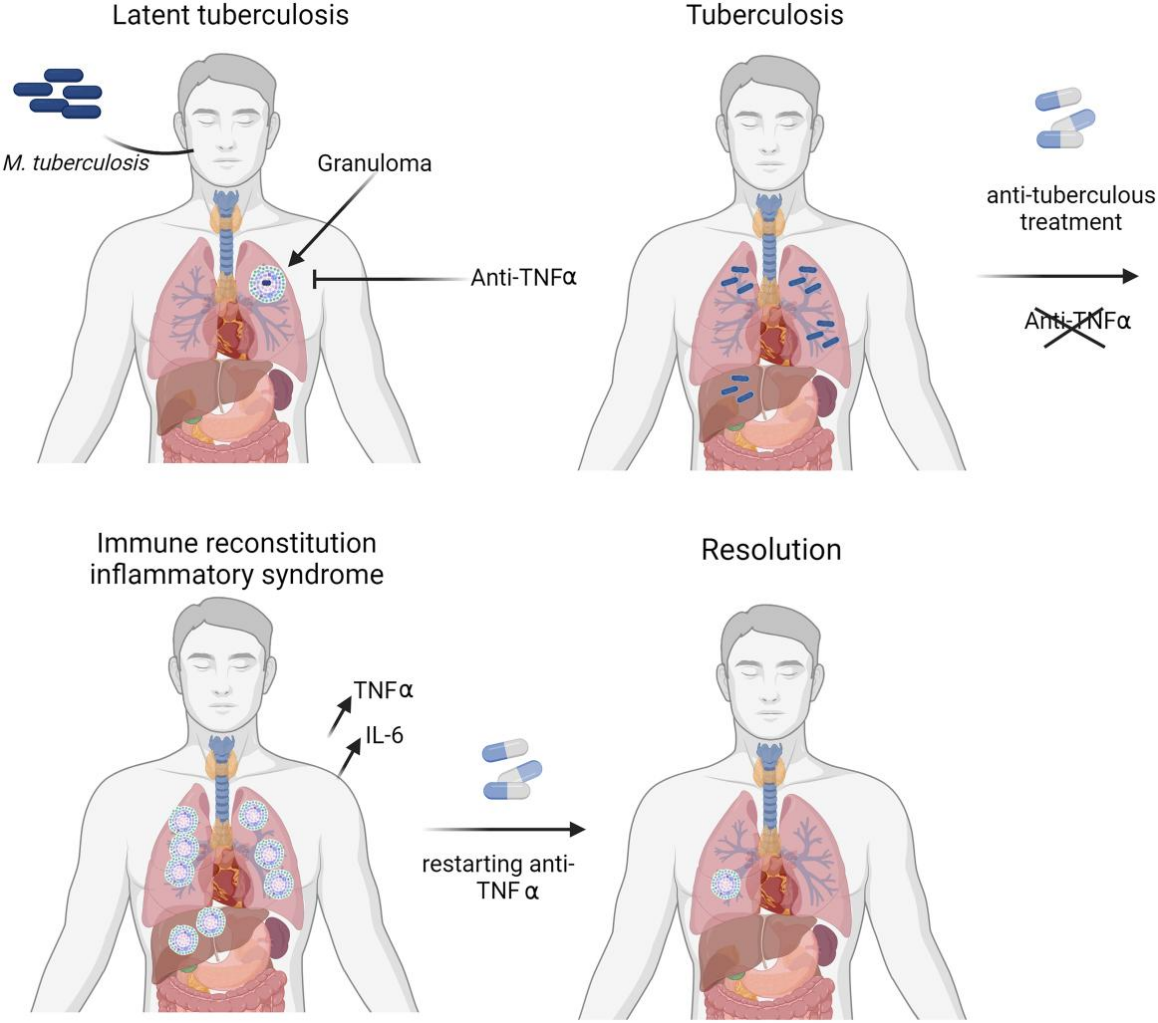
Proposed pathophysiology of immune reconstitution inflammatory syndrome complicating tuberculosis in a patient undergoing anti-TNFα therapy. Created with BioRender.com. anti-TNFα, anti–tumor necrosis factor α; IL-6, interleukin 6; *M tuberculosis*, *Mycobacterium tuberculosis*; TNFα, tumor necrosis factor α.

The role of TNFα in granuloma formation [22] and the studies showing that T lymphocytes produce TNFα in quantity during IRIS [23] provide pathophysiologic arguments for the use of these molecules in these indications (Figure 2). None of the patients in our cohort who had resumed anti-TNFα for digestive disease had IRIS after restarting treatment.

For patients in whom TB occurs under anti-TNF treatment, the fear is TB relapse. In our cohort, anti-TNFα was resumed in 21 cases and relatively quickly (median, 4 months), with only 1 case of TB relapse after anti-TNF resumption. Other studies had shown that it seemed reasonable to resume such treatment without the risk of relapse [20, 24], but the resumption was later than in our study (median, 12 and 11 months, respectively). A recent systematic literature review assessed the risk of TB relapse in patients who started or restarted TNF antagonists of JAK inhibitors. Of 368 patients with TB who initiated or reinitiated anti-TNF, only 14 (3.8%) relapsed after a median 8.5 months following the initiation/reinitiation [25]. Thus, it seems reasonable to restart treatment as soon as the bacillary load has been reduced by anti-TB treatment, either in the case of IBD activity requiring anti-TNF or in the case of symptomatic or severe IRIS.

Many patients in our study were originally from TB-endemic countries or had been exposed and developed active TB despite treated LTBI or negative screening. This raises the question of reexposure and how to proceed. Once anti-TNF treatment is started or after the treatment of initial LTBI, interferon γ release assays are no longer interpretable [7, 26]. It seems necessary to be particularly vigilant with these patients, providing information and preventive measures regarding the risk of TB and maintaining a perpetual concern about the occurrence of active TB.

Finally, our study has limitations, mainly due to its retrospective nature and the small number of patients, which makes generalization difficult. Because the study was retrospective and observational, we may have failed to collect important data, and some bias may have occurred. Because follow-up was not standardized, we may have missed late relapses, although the median follow-up after anti-TNFα resumption was 27 months (IQR, 17–82). However, the cases from the systematic literature review strongly support our results, even if there is a publication bias that cannot be separated from case reports. Randomized studies will be needed to draw definitive conclusions regarding the effectiveness and safety of anti-TNF treatment in TB-IRIS.

Overall, this work confirms that the presentation of TB in patients with IBD who are treated with anti-TNFα is often disseminated. It shows that IRIS is a common complication of varying severity. Anti-TNFα reintroduction appears to be a safe and effective strategy for the treatment of IRIS and IBD.

## Supplementary Material

ofae327_Supplementary_Data

## Supplementary Data

Supplementary materials are available at *Open Forum Infectious Diseases* online. Consisting of data provided by the authors to benefit the reader, the posted materials are not copyedited and are the sole responsibility of the authors, so questions or comments should be addressed to the corresponding author.

## Notes

***Author contributions.*** A. Amoura, T. F., and V. Z. contributed in study design, data collection, statistical analysis conduction and interpretation, and manuscript writing. All other authors contributed in data collection. A. Amoura is the guarantor of this study.

***Patient consent statement.*** This work was approved by the Ethics Committee of the French Infectiology Society (CER-MIT 2023-0904). The investigation conforms with the principles outlined in the Declaration of Helsinki. In accordance with the ethical standards of our hospital's institutional review board, the Committee for the Protection of Human Subjects, and French law, written informed consent was not needed for demographic, physiologic, and hospital outcome data analyses because this observational study did not modify existing diagnostic or therapeutic strategies; however, patients were informed of their inclusion in the study.

***Financial support*.** This study did not receive any financial support.
