# Correction to: Tuberculosis and Immune Reconstitution Inflammatory Syndrome in Patients With Inflammatory Bowel Disease and Anti-TNFα Treatment: Insights From a French Multicenter Study and Systematic Literature Review With Emphasis on Paradoxical Anti-TNFα Resumption

**DOI:** 10.1093/ofid/ofaf208

**Published:** 2025-04-08

This is a **correction** to:

Ariane Amoura, Thomas Frapard, Xavier Treton, Laure Surgers, Laurent Beaugerie, Matthieu Lafaurie, Jean Marc Gornet, Raphaël Lepeule, Aurélien Amiot, Etienne Canouï, Vered Abitbol, Antoine Froissart, Mathias Vidon, Yann Nguyen, Agnès Lefort, Virginie Zarrouk, Tuberculosis and Immune Reconstitution Inflammatory Syndrome in Patients With Inflammatory Bowel Disease and Anti-TNFα Treatment: Insights From a French Multicenter Study and Systematic Literature Review With Emphasis on Paradoxical Anti-TNFα Resumption, *Open Forum Infectious Diseases*, Volume 11, Issue 7, July 2024, ofae327, https://doi.org/10.1093/ofid/ofae327

An additional affiliation has been added for first and corresponding author, Ariane Amoura. This affiliation is: Sorbonne Université, Paris, France.

