# Supplementary material for: Tuberculosis and Immune Reconstitution Inflammatory Syndrome in Patients With Inflammatory Bowel Disease and Anti-TNFα Treatment: Insights From a French Multicenter Study and Systematic Literature Review With Emphasis on Paradoxical Anti-TNFα Resumption
Source: Open Forum Infect Dis. 2024 Jun 17;11(7):ofae327. doi: 10.1093/ofid/ofae327 (PMC11218776; doi:10.1093/ofid/ofae327)
Supplement: ofae327_Supplementary_Data [file ofae327_supplementary_data.docx]

**Supplementary documents**

**Title: Tuberculosis (TB) and immune reconstitution inflammatory syndrome (IRIS) in inflammatory bowel disease (IBD) patients on anti-TNFα treatment: insights from a French multicenter study and systematic literature review with emphasis on paradoxical anti TNFα resumption**

Ariane Amoura ^1,2^, MD ; Thomas Frapard ^3,4^, MD ; Xavier Treton ^5^, MD, PhD ; Laure Surgers ^6,7^ MD, PhD ; Laurent Beaugerie ^8^, MD, PhD ; Matthieu Lafaurie ^9^, MD ; Jean Marc Gornet ^10^, MD, PhD ; Raphaël Lepeule ^11^, MD ; Aurélien Amiot ^12^, MD, PhD ; Etienne Canoui ^13^, MD ; Vered Abitbol ^14^, MD, PhD ; Antoine Froissart ^15^, MD ; Mathias Vidon ^16^, MD ; Yann Nguyen ^1,17^, MD, PhD ; Agnès Lefort ^1,2^, MD, PhD ; Virginie Zarrouk ^1^, MD

Corresponding author: Dr Ariane Amoura, MD, Service de Médecine Interne, Hôpital Beaujon, 100 boulevard du Général Leclerc, 92110 Clichy, France, [ariane.amoura@aphp.fr](mailto:ariane.amoura@aphp.fr)

Alternate corresponding author: Dr Virginie Zarrouk, MD, Service de Médecine Interne, Hôpital Beaujon, 100 boulevard du Général Leclerc, 92110 Clichy, France, virginie.zarrouk@aphp.fr

**Affiliations**

1 Service de Médecine Interne, Hôpital Beaujon, Assistance Publique des Hôpitaux de Paris (AP-HP), Clichy, France

2 Groupe de recherche Infection Antimicrobials Modelling Evolution (IAME), Inserm U1137, Université Paris Cité, Paris, France.

3 Service de Médecine Intensive et Réanimation, Hôpital Henri Mondor, DHU ATVB, AP-HP, Créteil, France

4 Université Paris Est Créteil, Faculté de Médecine de Créteil, Institut Mondor de Recherche Biomédicale-Groupe de Recherche Clinique CARMAS, Créteil, France

5 Institut des MICI, Groupe hospitalier privé Ambroise-Paré- Hartmann, Neuilly, France

6 Service des Maladies Infectieuses et Tropicales, Hôpital Saint-Antoine, AP-HP. Sorbonne Université, France

7 Sorbonne Université, INSERM, Institut Pierre Louis d’Épidémiologie et de Santé Publique, F75012, Paris, France

8 Service de Gastroentérologie, Hôpital Saint Antoine, AP-HP, Paris, France

9 Service de Maladies infectieuses et Tropicales, Hôpital Saint-Louis-Hôpital Lariboisière, AP-HP, Paris, France.

10 Service de Gastroentérologie, Hôpital Saint-Louis-Hôpital, AP-HP, Paris, France.

11 Unité Transversale de Traitement des Infections, AP-HP, Hôpitaux Universitaires Henri Mondor, Créteil, F-94010, France.

12 Service de Gastroentérologie, Hôpitaux Universitaires Henri Mondor, AP-HP, Créteil, France.

13 Équipe Mobile d'Infectiologie, AP-HP, APHP.CUP, Hôpital Cochin, F-75014 Paris, France.

14 Service de gastroentérologie, Hôpital Cochin, AP-HP, Université Paris Cité, Paris, 75014, France.

15 Service de Médecine interne, Centre Hospitalier Intercommunal de Créteil, Créteil, France.

16 Service de Gastroentérologie, Centre Hospitalier Intercommunal de Créteil, Créteil, France.

17 Centre de recherche en immunologie des maladies, INSERM U1184, Université Paris Saclay, Le Kremlin-Bicêtre, France

## Pubmed

| **Terms** | **Results** |
| --- | --- |
| ((tuberculosis) OR (TB)) AND ((anti TNF) OR (infliximab) OR (adalimumab) OR (etanercept) OR (golimumab) OR (Certolizumab)) AND ((IRIS) OR (immune reconstitution inflammatory syndrome) OR (paradoxical tuberculosis) OR (paradoxical TB) OR (PR) OR (paradoxical reaction)) | **123** |

## Embase

| **#** | **Terms** | **Results** |
| --- | --- | --- |
| 1 | Tuberculosis | 394538 |
| 2 | TB | 110882 |
| 3 | 1 OR 2 | 432529 |
| 4 | Anti TNF alpha | 99103 |
| 5 | Infliximab | 64182 |
| 6 | Adalimumab | 46380 |
| 7 | Etanercept | 30564 |
| 8 | Certolizumab | 10251 |
| 9 | Golimumab | 9960 |
| 10 | 4 OR 5 OR 6 OR 7 OR 8 OR 9 | 178387 |
| 11 | IRIS | 67293 |
| 12 | Immune reconstitution inflammatory syndrome | 4994 |
| 13 | Paradoxical tuberculosis | 1074 |
| 14 | Paradoxical TB | 386 |
| 15 | 11 OR 12 OR 13 OR 14 | 71345 |
| 16 | 3 AND 10 AND 15 | **213** |

## Ovid

| **#** | **Terms** | **Results** |
| --- | --- | --- |
| 1 | Tuberculosis | 269991 |
| 2 | TB | 59458 |
| 3 | 1 OR 2 | 286720 |
| 4 | Anti TNF alpha | 11228 |
| 5 | Infliximab | 15579 |
| 6 | Adalimumab | 9306 |
| 7 | Etanercept | 8766 |
| 8 | Certolizumab | 1311 |
| 9 | Golimumab | 1355 |
| 10 | 4 OR 5 OR 6 OR 7 OR 8 OR 9 | 31984 |
| 11 | IRIS | 27264 |
| 12 | Immune reconstitution inflammatory syndrome | 1772 |
| 13 | Paradoxical tuberculosis | 41 |
| 14 | Paradoxical TB | 27 |
| 15 | 11 OR 12 OR 13 OR 14 | 28192 |
| 16 | 3 AND 10 AND 15 | **30** |

COCHRANE

| #1 | tuberculosis | 8590 |
| --- | --- | --- |
| #2 | TB | 7864 |
| #3 | #1 OR #2 | 13489 |
| #4 | anti TNF | 5421 |
| #5 | infliximab | 2761 |
| #6 | adalimumab | 3944 |
| #7 | etanercept | 2488 |
| #8 | golimumab | 829 |
| #9 | Certolizumab | 817 |
| #10 | #4 OR #5 OR #6 OR #7 OR #8 OR #9 | 12694 |
| #11 | IRIS | 1698 |
| #12 | immune reconstitution inflammatory syndrome | 183 |
| #13 | paradoxical Tuberculosis | 47 |
| #14 | paradoxical TB | 39 |
| #15 | paradoxical reaction | 143 |
| #16 | #11 OR #12 OR #13 OR #14 OR #15 | 1946 |
| #17 | #3 AND #10 AND #16 | **6** |

**Supplementary table 1:** Search strategy for systematic literature review

| **Study** | **Number cases TB under anti TNF treatment + IRIS** | **Number cases TB-IRIS or TB-PR treated with anti TNF** |
| --- | --- | --- |
| Wallis et al, 2009^27^ | 1 | 1 |
| Lee et al, 2014^28^ | 1 | 0 |
| Dussouillez et al, 2020^29^ | 1 | 0 |
| Abousnar et al, 2009^30^ | 1 | 0 |
| Roche et al, 2019^31^ | 1 | 0 |
| Hosomi et al, 2022^32^ | 1 | 0 |
| Strady et al, 2006^33^ | 1 | 0 |
| Hess et al, 2011^34^ | 1 | 0 |
| Jorge et al, 2012^35^ | 1 | 1 |
| Arend et al, 2007^36^ | 1 | 0 |
| Moreno et al, 2023^37^ | 1 | 1 |
| Tanaka et al, 2015^38^ | 1 | 0 |
| Christian et al, 2022^39^ | 1 | 0 |
| Benchoukroun et al, 2020^40^ | 1 | 0 |
| Unlu et al, 2014^41^ | 1 | 0 |
| Marino et al, 2010^42^ | 1 | 0 |
| Piffer et al, 2019^43^ | 1 | 0 |
| Kassapidis et al, 2020^44^ | 1 | 0 |
| Hristea et al, 2014^45^ | 1 | 0 |
| Samuel et al, 2017^46^ | 1 | 0 |
| Rivoisy et al, 2016^11^ | 14 | 0 |
| Yoon et al, 2009^47^ | 1 | 0 |
| Garcia Vidal et al, 2005^12^ | 4 | 0 |
| Moureau et al, 2012^48^ | 1 | 0 |
| Watanabe et al, 2017^17^ | 1 | 0 |
| Szerszen et al, 2009^49^ | 1 | 0 |
| Falkenstern Ge et al, 2015^50^ | 2 | 0 |
| Belkhir et al, 2010^51^ | 1 | 0 |
| Saez-Gonzalez et al, 2019^52^ | 1 | 0 |
| Armange et al, 2023^14^ | 1 | 3 |
| O Dowd et al, 2011^53^ | 1 | 1 |
| Tani et al, 2018^54^ | 1 | 0 |
| Nabeya et al, 2020^55^ | 1 | 0 |
| Belknap et al, 2005^56^ | 1 | 0 |
| Vandecasteele et al, 2012^57^ | 1 | 1 |
| Lee et al, 2012^58^ | 0 | 1 |
| Hsu et al, 2016^59^ | 0 | 2 |
| Lwin et al, 2018^60^ | 0 | 1 |
| Richaud et al, 2015^61^ | 0 | 1 |
| Onifade et al, 2011^62^ | 0 | 1 |
| Azeem et al, 2021^63^ | 0 | 1 |
| Abo et al, 2021^64^ | 0 | 4 |
| Molton et al, 2015^65^ | 0 | 2 |
| Goulenok et al, 2022^66^ | 0 | 1 |
| Faiz et al, 2018^67^ | 0 | 1 |
| Eshagh et al, 2020^68^ | 0 | 1 |
| Kim et al, 2021^69^ | 0 | 1 |
| Chia et al, 2022^18^ | 0 | 1 |
| Briner et al, 2021^70^ | 0 | 1 |
| Storms et al, 2019^71^ | 0 | 1 |
| Blackmore et al, 2008^72^ | 0 | 1 |
| Santin et al, 2020^73^ | 0 | 2 |
| Laparra et al, 2021^74^ | 0 | 5 |
| Marais et al, 2020^75^ | 0 | 4 |

**Supplementary table 2:** list of studies included in the systematic literature review

**References**

1. Wallis RS, van Vuuren C, Potgieter S. Adalimumab treatment of life-threatening tuberculosis. Clin Infect Dis Off Publ Infect Dis Soc Am. 2009 May 15;48(10):1429–32.

2. Lee B, Moosavy F. Pulmonary Embolism following Cessation of Infliximab for Treatment of Miliary Tuberculosis. Case Rep Pulmonol. 2014;2014:479025.

3. Dussouillez G, Zayet S, Kone D, Royer PY, Toko-Tchuindzie L, Ruyer O, et al. [Tuberculous liver abscess, anti-TNF alpha, and immune reconstitution inflammatory syndrome]. Med Mal Infect. 2020 Feb;50(1):90–1.

4. Abounasr KK, Rogers L. A CASE OF IMMUNE RECONSTITUTION SYNDROME SECONDARY TO DISCONTINUATION OF ANTI-TNF AGENTS IN A PATIENT WITH PULMONARY TUBERCULOSIS. CHEST. 2009 Oct 1;136(4):51S.

5. Roche S, McLaughlin A m., Keane J m. A Case of Multiple Intracranial Tuberculomas as a Presentation of Tuberculosis Immune Reconstitution Inflammatory Syndrome (TB IRIS) in a Patient with Mycobacterium Bovis Tuberculosis. In: C53 TUBERCULOSIS CASE REPORTS [Internet]. American Thoracic Society; 2019 [cited 2023 Aug 25]. p. A5118–A5118. (American Thoracic Society International Conference Abstracts). Available from: https://www.atsjournals.org/doi/abs/10.1164/ajrccm-conference.2019.199.1_MeetingAbstracts.A5118

6. Hosomi S, Sugita N, Kanamori A, Ominami M, Otani K, Kamata N, et al. A case of paradoxical response during anti-tuberculosis treatment in a patient with ulcerative colitis. Clin J Gastroenterol. 2022 Jun;15(3):592–7.

7. Strady C, Brochot P, Ainine K, Jegou J, Remy G, Eschard JP, et al. Tuberculose lors d’un traitement par agents inhibiteurs du TNF alpha. Presse Médicale. 2006 Nov 1;35(11, Part 2):1765–72.

8. Hess S, Hospach T, Nossal R, Dannecker G, Magdorf K, Uhlemann F. Life-threatening disseminated tuberculosis as a complication of TNF-α blockade in an adolescent. Eur J Pediatr. 2011 Oct;170(10):1337–42.

9. Jorge JH, Graciela C, Pablo AP, Luis SHJ. A life-threatening central nervous system-tuberculosis inflammatory reaction nonresponsive to corticosteroids and successfully controlled by infliximab in a young patient with a variant of juvenile idiopathic arthritis. J Clin Rheumatol Pract Rep Rheum Musculoskelet Dis. 2012 Jun;18(4):189–91.

10. Arend SM, Leyten EMS, Franken WPJ, Huisman EM, van Dissel JT. A patient with de novo tuberculosis during anti-tumor necrosis factor-alpha therapy illustrating diagnostic pitfalls and paradoxical response to treatment. Clin Infect Dis Off Publ Infect Dis Soc Am. 2007 Dec 1;45(11):1470–5.

11. Moreno Y b., Cintron M, Hernandez F. An Unusual Diffused Tuberculosis Infection Associated With Tuberculosis-Immune Reconstitution Inflammatory Syndrome in a Non-HIV Patient. In: B63 IMMUNOCOMPROMISED HOST PROBLEMS AND COMPLICATIONS [Internet]. American Thoracic Society; 2023 [cited 2023 Sep 25]. p. A3897–A3897. (American Thoracic Society International Conference Abstracts). Available from: https://www.atsjournals.org/doi/abs/10.1164/ajrccm-conference.2023.207.1_MeetingAbstracts.A3897

12. Tanaka T, Sekine A, Tsunoda Y, Takoi H, Lin SY, Yatagai Y, et al. Central nervous system manifestations of tuberculosis-associated immune reconstitution inflammatory syndrome during adalimumab therapy: a case report and review of the literature. Intern Med Tokyo Jpn. 2015;54(7):847–51.

13. Christian E, Johnston A. CNS TB-IRIS Following Cessation of Adalimumab in an Adolescent With Crohn’s Disease. Open Forum Infect Dis. 2022 Aug 1;9(8):ofac367.

14. Benchoukroun S, Gaillard S, Vermersch-Langlin A, Deregnaucourt D. Disseminated tuberculosis complicated by immune reconstitution inflammatory syndrome under anti-TNF treatment. Eur J Dermatol EJD. 2020 Jun 1;30(3):322–3.

15. Unlu M, Cimen P, Ayranci A, Akarca T, Karaman O, Dereli MS. Disseminated tuberculosis infection and paradoxical reaction during antimycobacterial treatment related to TNF-alpha blocker agent Infliximab. Respir Med Case Rep. 2014;13:43–7.

16. Troncoso Mariño A, Campelo Sánchez E, Martínez López de Castro N, Inaraja Bobo MT. Haemophagocytic syndrome and paradoxical reaction to tuberculostatics after treatment with infliximab. Pharm World Sci PWS. 2010 Apr;32(2):117–9.

17. Piffer F, Levi G, Marchetti G, Barbieri C. Immune reconstitution inflammatory syndrome in tuberculous pleurisy and ulcerative colitis: a case report. Monaldi Arch Chest Dis Arch Monaldi Mal Torace. 2019 Sep 10;89(3).

18. Kassapidis V, Adelman M. IMMUNE RECONSTITUTION INFLAMMATORY SYNDROME: THE RISK OF ADALIMUMAB CESSATION IN TB TREATMENT. CHEST. 2020 Oct 1;158(4):A526.

19. Hristea A, Munteanu D, Jipa R, Mihăilescu R, Manea E, Hrişcă R, et al. IRIS associated with tuberculosis of CNS in HIV and non-HIV infected patients: how long do we need to use steroids. BMC Infect Dis. 2014 Oct 15;14(Suppl 7):P42.

20. Samuel G, Storey D, Singh D, Zhou XJ. Membranoproliferative Glomerulonephritis Secondary to Disseminated Tuberculosis After Withdrawal of Adalimumab. In: D25 UNUSUAL INFECTIONS: CASE REPORTS [Internet]. American Thoracic Society; 2017 [cited 2023 Oct 6]. p. A7168–A7168. (American Thoracic Society International Conference Abstracts). Available from: https://www.atsjournals.org/doi/abs/10.1164/ajrccm-conference.2017.195.1_MeetingAbstracts.A7168

21. Rivoisy C, Tubach F, Roy C, Nicolas N, Mariette X, Salmon D, et al. Paradoxical anti-TNF-associated TB worsening: Frequency and factors associated with IRIS. Joint Bone Spine. 2016 Mar;83(2):173–8.

22. Yoon YK, Kim JY, Sohn JW, Kim MJ, Koo JS, Choi JH, et al. Paradoxical response during antituberculous therapy in a patient discontinuing infliximab: a case report. J Med Case Reports. 2009 Apr 1;3:6673.

23. Garcia Vidal C, Rodríguez Fernández S, Martínez Lacasa J, Salavert M, Vidal R, Rodríguez Carballeira M, et al. Paradoxical response to antituberculous therapy in infliximab-treated patients with disseminated tuberculosis. Clin Infect Dis Off Publ Infect Dis Soc Am. 2005 Mar 1;40(5):756–9.

24. Moureau C, Pothen L, Wilmes D, Yombi JC, Coche E, Hainaut P. Paradoxical response to tuberculosis treatment in a patient receiving tumor necrosis factor-alpha antagonist. Am J Med. 2012 Jun;125(6):e9–10.

25. Watanabe S, Kaneko Y, Kawamoto H, Maehara T, Baba Y, Fujisaki I, et al. Paradoxical response with increased tumor necrosis factor-α levels to anti-tuberculosis treatment in a patient with disseminated tuberculosis. Respir Med Case Rep. 2017;20:201–4.

26. Szerszen A, Gupta S, Seminara D, Jarrett M, Goldstein M. Peritoneal tuberculosis complicated by immune reconstitution inflammatory syndrome in a patient treated with infliximab?: a case for adjuvant immunosuppressive therapy. J Clin Rheumatol Pract Rep Rheum Musculoskelet Dis. 2009 Dec;15(8):417–8.

27. Falkenstern-Ge RF, Husemann K, Kohlhäufl M. Prolonged paradoxical reaction to anti-tuberculous treatment after discontinuation of TNF-alpha- blocker therapy with adalimumab. Rare clinical documentation. Open Med Wars Pol. 2015;10(1):39–43.

28. Melboucy-Belkhir S, Flexor G, Stirnemann J, Morin AS, Boukari L, Polliand C, et al. Prolonged paradoxical response to anti-tuberculous treatment after infliximab. Int J Infect Dis IJID Off Publ Int Soc Infect Dis. 2010 Sep;14 Suppl 3:e333-334.

29. Sáez-González E, Salavert M, Cerrillo E, Moret I, Iborra M, Nos P, et al. Secondary Haemophagocytic Syndrome and Overlapping Immune Reconstitution Syndrome: Life-Threatening Complications of Anti-TNF-α Treatment for Crohn’s Disease. Am J Gastroenterol. 2019 Jan;114(1):177–9.

30. Armange L, Lacroix A, Petitgas P, Arvieux C, Piau-Couapel C, Poubeau P, et al. The use of TNF-α antagonists in tuberculosis to control severe paradoxical reaction or immune reconstitution inflammatory syndrome: a case series and literature review. Eur J Clin Microbiol Infect Dis Off Publ Eur Soc Clin Microbiol. 2023 Apr;42(4):413–22.

31. O’Dowd C, Kewin P, Morris J, Cotton M. Tuberculosis complicated by immune reconstitution inflammatory syndrome in a patient on anti-TNFα therapy for Crohn’s disease. BMJ Case Rep. 2011 Mar 8;2011.

32. Tani Y, Miyamae T, Hara T, Yamanaka H. Tuberculosis-immune reconstitution inflammatory syndrome associated with juvenile enthesitis-related arthritis under treatment of adalimumab. Mod Rheumatol Case Rep. 2018 Jul 3;2(2):149–52.

33. Nabeya D, Kinjo T, Yamaniha K, Yamazato S, Tome R, Miyagi K, et al. Use of steroids to treat anti-tumor necrosis factor α induced tuberculosis-associated immune reconstitution inflammatory syndrome. Medicine (Baltimore) [Internet]. 2020 Oct 23 [cited 2021 Apr 23];99(43). Available from: https://www.ncbi.nlm.nih.gov/pmc/articles/PMC7581145/

34. Belknap R, Reves R, Burman W. Immune reconstitution to Mycobacterium tuberculosis after discontinuing infliximab. Int J Tuberc Lung Dis Off J Int Union Tuberc Lung Dis. 2005 Sep;9(9):1057–8.

35. Vandecasteele SJ, De Vriese AS, Vanhooren GT. Reversion of cerebral artery stenoses due to tuberculomas with TNF-α antibodies. Clin Neurol Neurosurg. 2012 Sep;114(7):1016–8.

36. Adalimumab treatment may replace or enhance the activity of steroids in steroid-refractory tuberculous meningitis | SpringerLink [Internet]. [cited 2023 Oct 6]. Available from: https://link.springer.com/article/10.1007/s10156-011-0334-y

37. Hsu DC, Faldetta KF, Pei L, Sheikh V, Utay NS, Roby G, et al. A Paradoxical Treatment for a Paradoxical Condition: Infliximab Use in Three Cases of Mycobacterial IRIS. Clin Infect Dis Off Publ Infect Dis Soc Am. 2016 Jan 15;62(2):258–61.

38. Lwin N, Boyle M, Davis JS. Adalimumab for Corticosteroid and Infliximab-Resistant Immune Reconstitution Inflammatory Syndrome in the Setting of TB/HIV Coinfection. Open Forum Infect Dis. 2018 Feb;5(2):ofy027.

39. Richaud C, Ghosn J, Amazzough K, Poiree S, Lortholary O. Anti-tumor necrosis factor monoclonal antibody for steroid-dependent TB-IRIS in AIDS. AIDS Lond Engl. 2015 Jun 1;29(9):1117–9.

40. Onifade D, Logan S, Marshall N, Lipman M, Bhagani S, Cropley I. Immunomodulatory therapy in the management of paradoxical progression of tuberculomas in patients at Royal Free Hospital. J Infect. 2011;63(6):e111–2.

41. Azeem A, Ahmad F, Velagapudi M. 1401. Infliximab for Immune Reconstitution Inflammatory Syndrome (IRIS) in Tuberculous Meningitis; A Treatment Paradox. Open Forum Infect Dis. 2021 Nov 1;8(Supplement_1):S785.

42. Abo YN, Curtis N, Butters C, Rozen TH, Marais BJ, Gwee A. Successful Treatment of a Severe Vision-Threatening Paradoxical Tuberculous Reaction with Infliximab: First Pediatric Use. Pediatr Infect Dis J. 2020 Apr;39(4):e42–5.

43. Molton JS, Huggan PJ, Archuleta S. Infliximab therapy in two cases of severe neurotuberculosis paradoxical reaction. Med J Aust. 2015 Feb 16;202(3):156–7.

44. Goulenok T, Gaudemer A, Rouzaud D, Chauveheid MP, Alexandra JF, Sacre K, et al. Infliximab to Treat Severe Paradoxical Reaction in HIV-Negative Tuberculous Meningoencephalitis. Neurology. 2022 Jan 18;98(3):118–9.

45. Faiz S, Ettahar N, Sabbah M, Toné A, Benrezkallah M, Bourgarit A, et al. Traitement par infliximab d’une réaction paradoxale neuroméningée chez un patient immunocompétent compliquant une tuberculose ganglionnaire, pulmonaire et méningée. Médecine Mal Infect. 2018 Oct 1;48(7):484–7.

46. Eshagh D, Benali K, Dossier A, Chauveheid MP, Rouzaud D, Goulenok T, et al. Infliximab use for corticosteroid-resistant tuberculosis immune reconstitution inflammatory syndrome (TB-IRIS) in an immunocompetent patient. Infection. 2020 Oct;48(5):799–802.

47. Kim KW, Kim HJ, Kim HW, Kim SH, Lee SA, Koo YS. Intractable Tuberculous Meningitis With Paradoxical Reactions Treated by Anti–Tissue Necrosis Factor-α Therapy. Neurol Clin Pract. 2021 Aug;11(4):e555–7.

48. Chia G, Bartlett T, Towns C, Blackmore T. Neurotuberculosis with paradoxical reaction treated with infliximab: case report and literature review. N Z Med J. 2022 Aug 5;135(1559):118–21.

49. Briner M, Oberholzer M, Chan A, Wagner F. Potential disease trigger as a therapeutic option: infliximab for paradoxical reaction in tuberculosis of the central nervous system. BMJ Case Rep. 2021 Aug 2;14(8):e235511.

50. Storms I, Magis-Escurra C, Hoefsloot W. Severe Paradoxical Reaction in a 20 Year Old Woman with Disseminated Tuberculosis. In: C53 TUBERCULOSIS CASE REPORTS [Internet]. American Thoracic Society; 2019 [cited 2023 Sep 25]. p. A5110–A5110. (American Thoracic Society International Conference Abstracts). Available from: https://www.atsjournals.org/doi/abs/10.1164/ajrccm-conference.2019.199.1_MeetingAbstracts.A5110

51. Blackmore TK, Manning L, Taylor WJ, Wallis RS. Therapeutic use of infliximab in tuberculosis to control severe paradoxical reaction of the brain and lymph nodes. Clin Infect Dis Off Publ Infect Dis Soc Am. 2008 Nov 15;47(10):e83-85.

52. Santin M, Escrich C, Majòs C, Llaberia M, Grijota MD, Grau I. Tumor necrosis factor antagonists for paradoxical inflammatory reactions in the central nervous system tuberculosis: Case report and review. Medicine (Baltimore). 2020 Oct 23;99(43):e22626.

53. Laparra A, Lerolle N, Gerin M, Cheret A, De Castro N, Gallien S, et al. Tumor necrosis factor-alpha inhibitors for extended or severe neurological immune reconstitution inflammatory syndrome in HIV-infected patients. AIDS. 2021 May 1;35(6):995.

54. Marais BJ, Cheong E, Fernando S, Daniel S, Watts MR, Berglund LJ, et al. Use of Infliximab to Treat Paradoxical Tuberculous Meningitis Reactions. Open Forum Infect Dis. 2021 Jan;8(1):ofaa604.
